# State of the ant: how broad is our recent knowledge of Neotropical ant behavior?

**DOI:** 10.3389/finsc.2025.1613264

**Published:** 2025-09-10

**Authors:** Rosannette Quesada-Hidalgo, Yorlenis González, Dumas Gálvez, Peter R. Marting, Armando Castillo-Pimentel, Jane Aguilar, Stephen Cox, Carrie Smith, Sabrina Amador-Vargas

**Affiliations:** ^1^ Smithsonian Tropical Research Institute (STRI), Panama City, Panama; ^2^ Sistema Nacional de Investigación (SNI), Panama City, Panama; ^3^ Estación Científica Coiba AIP, Panama City, Panama; ^4^ Centro de Neurociencias, Instituto de Investigaciones Científicas y Servicios de Alta Tecnología (INDICASAT AIP), Panama City, Panama; ^5^ Natural & Physical Sciences Libraries, Smithsonian Libraries and Archives, Washington, DC, United States

**Keywords:** formicidae, Neotropical ants, geographical bias, taxonomic bias, ant review

## Abstract

Over the years, most scholarly published papers have studied vertebrates, despite invertebrates’ higher species diversity and number of individuals. This has led to an inaccurate representation of global biodiversity patterns in scientific publications. Furthermore, the bias for studying vertebrates is also evident when comparing studies conducted in the tropics vs. temperate zones. We investigated whether similar taxonomic and geographical biases are maintained in recent years when studying the behavior of Neotropical ants. We searched for papers published between 2015 and 2022 with the words “ant” OR “ants” OR formicidae; tropic* OR neotropic* and behav* AND tropic* OR neotropic*. We found that recently published papers studying ants cover only ~10% of the Neotropical ant species, with a primary focus on economically damaging and/or invasive ants. Our results revealed that studies on ant behavior in the Neotropics are dominated by four species, which represent less than 0.15% of the ant species in the Neotropics, and that 50% of the focal species were mentioned only once or twice in studies regarding behavior. Moreover, recent ant behavior studies cover only approximately 8% of the Neotropical ant biodiversity. We found that the Neotropical countries where most ants have been collected for behavioral studies are Brazil, Panama, and Costa Rica. In contrast, other Central American countries are absent from the recent ant literature. Our results reveal concerning patterns of taxonomic and geographical inequity in the study of Neotropical ant behavior, despite its potential role in managing ant invasions and ensuring effective conservation measures. We highlight the need to broaden behavioral studies in the Neotropics and urge researchers to investigate relatively unknown ant species, and include understudied countries with limited scientific resources to fill this critical gap in current ant research.

## Introduction

Biology seeks a better comprehension of patterns in nature. These patterns could be more easily deciphered if our studies included a broader taxonomic representation. Therefore, if studying an extensive array of organisms facilitates an understanding of the rules of life, we expect the number of scientific studies from each taxonomic group to scale proportionally with its biodiversity (1). However, it is not surprising that some organisms are studied more than others. Over the last few years, invertebrates (mainly insects and arachnids), were represented in half of the biodiversity studies, despite accounting for 95% of all described animal species (2). Moreover, there is a similar strong bias toward endothermic vertebrates and against arthropods in animal behavior studies. Arthropods represent 84% of all animal species, but appear in only 27% of the publications, while birds and mammals, which represent less than 1.5% of the animal species diversity, account for more than half of the scientific records of behavioral studies (1). Additionally, the bias against invertebrates tends to be greater in highly cited papers (2).

Given that the tropics are the most biodiverse region of the world, both in terms of abundance and species richness (3), the corresponding pattern would also be expected when examining scientific records, that is, a higher number of studies taking place in the tropics compared to temperate zones. However, tropical countries tend to have fewer biodiversity studies despite being more biodiverse than temperate regions (2), and even within the tropics, the geographical distribution of research is unequal, taking place in only a few countries (4). Moreover, the taxonomic bias toward studying vertebrates is greater in the tropics (2).

The reasons behind these taxonomic and geographical patterns in scientific research vary. Larger animals, especially mammals, are generally perceived as more charismatic and preferred by both adults and children in zoo exhibits (5). Another reason is that identifying insects is much more challenging than identifying vertebrates, particularly at the species level, and usually requires the expertise of a taxonomist. The difficulty in identifying invertebrates is further exacerbated by their immense biodiversity, especially in the tropics (6). For instance, on a single tree in the rainforest, there are more species of ants than in the entire country of England (7). A third reason explaining these biases may be a taxonomic impediment, as the number of properly trained taxonomists and taxonomic specialists is decreasing, likely because of limited financial resources such as grants and positions in taxonomy (8, 9). The consequences of this taxonomy crisis can lead to fewer researchers studying megadiverse groups of organisms (9), and an underrepresentation of the world’s described biodiversity and its phylogenetic relationships, which may have an outsized effect on developing countries in the tropics (9, 10).

Ant taxonomy is relatively well studied compared to other arthropod groups, at least to the genus level (11, 12). Moreover, all ants are social, and, similar to humans, need to solve problems related to living in societies, which makes them ideal for investigating the behavioral strategies involving traffic (e.g., 13), trail design (e.g., 14), waste management (15), and epidemics (16, 17). Some invasive ant species are responsible for severe negative socioeconomic and environmental impacts worldwide (18). Invasive ant colonies can reduce the biodiversity or abundance of native ant communities, disrupting mutualistic relationships (19), with harmful consequences to local ant species and other native invertebrates, vertebrates, and plants (20). Furthermore, invasive species can cause significant damage to agriculture, public health, and social welfare (21). Last, some ant species even within their native range are considered pests (e.g., leaf-cutting ants; 22, 23). Despite their relevance, ants remain underrepresented in behavioral studies (1).

Given the importance of studying ant behavior and the marked bias against studying invertebrates in the tropics, we investigated how the number of recently published scientific studies examining Neotropical ant behavior compares to the current reported diversity of Neotropical ant species. We defined behavior broadly, encompassing all observable actions and responses to stimuli as ants interact with each other and their physical environment. Therefore, some papers categorized here under “behavior” may include ecological aspects, but according to our broad criteria, they were included as long as they advance our understanding of ant behavior. We searched for research published in the eight-year period between 2015 and 2022 to shed light on recent taxonomical and geographical patterns in the study of ants. We also determined whether ant behavior was mainly studied in the field, under laboratory conditions, or as theoretical simulations or modeling. We compared whether the number of recent studies of ants was proportional to the diversity of recorded species per subfamily. Finally, we determined the number of reports from each country. The results of this research may help redirect future research efforts toward studying new behaviors from uncommonly studied ants within the Neotropics and detect possible future ant invasions or potential pests.

## Materials and methods

We used two search approaches to examine published papers regarding ants: a general search to capture the most records of published papers about ants (only from 2015 to 2019), and a complementary targeted search (from 2015 to 2022), where we examined papers from specific scientific journals for detailed information.

For the general search, we searched in 6 databases (Web of Science, Zoological Record, SciELO, PubMed, ProQuest Databases, and Google Scholar; we excluded Elsevier’s Scopus platform due to its overlap in indexed journals with Clarivate’s Web of Science, and used Web of Science given that it aligns more with subjects such as conservation biology, ecology, environmental science, veterinary science, and zoology) and performed 3 baseline searches on each database: A) (“ant” OR “ants” OR formicidae); B) (tropic* OR neotropic*) and; C) (behav* AND (tropic* OR neotropic*) (**Supplementary Table S1**). We then performed two complex search strings: 1) searches A&B combined and 2) searches A&C combined. This general search provides a broader understanding of how many ant research papers published in recent years address the behavior of Neotropical ants, as it is not limited to specific journals. The general research was conducted only for papers published between 2015 and 2019.

The targeted search, which provides detailed information about the studied ant species and collection sites, was conducted for papers published between 2015 and 2022. Our aim was to assess the state of the recently published research regarding the behavior of Neotropical ants. We focused on an eight-year period due to possible recent methodological advances, changes in research priorities, and the availability of comparable data during this timeframe. We consider a five-year period as recent, and added a buffer of three years in case any outlier papers exist to obtain an up-to-date dataset.

The papers included in this search were manually curated to understand whether behavioral information was included or not. More specifically, for the targeted search, we selected 23 journals (**Supplementary Table S2**) with a wide range of impact factors, covering various subjects in biology, including ecology, behavior, and social insects. Using Web of Science (All databases), we examined for the words “ants” OR “ant” OR “Formicidae”. We filtered our search by ‘name of publication’, where we wrote the name of each selected journal as in **Supplementary Table S2**, and set the publication period to be between 2015 and 2022. We then limited each journal search to ‘journal articles’ only. Because we were focused on the behavior of ants with a Neotropical distribution, for each paper retrieved on this search, we first recorded whether the studied species had a tropical, Neotropical, or temperate distribution, based on distribution maps at Antmaps (https://antmaps.org/; 24, 25), and information in 26 (Accessed between September 10th, 2021 and October 2nd, 2023). We classified species as “tropical” if the ants were collected or observed in a tropical country or if at least part of the distribution of the ant species was in the tropics. We defined tropics as the area between the tropics of Cancer (23° 26′ 11.4” N) and Capricorn (23° 26′ 11.4” S), and as “Neotropical” those tropical ants that were collected or observed within those latitudes in the American continent and Caribbean islands. We classified species as “temperate” if their distribution was not within the tropics. We then recorded if the paper studied any aspect of ant behavior. Our criteria for including a paper as “behavior” were broad, and we even included papers where the behavioral information was only a brief section of the paper. Topics that we considered as contributing to our understanding of ant behavior included navigation and homing, communication, colony dispersion, foraging, predation, decision-making, natural history, the evolution of behaviors (i.e., mapping behaviors onto phylogenies), and social structure. Papers not classified as studying behavior included topics such as morphology (if the paper did not study a behavior related to that morphology), taxonomy, phylogeny, physiology, chemistry, genetics, and distribution.

We recorded all studied ant species and genera mentioned in each retrieved paper (using the scientific name provided by the authors), and documented the ant subfamily, following the taxonomical classification in Antwiki (https://www.antwiki.org/, accessed between September 10th, 2021 and October 2nd, 2023: 24, 25). Subspecies were recorded separately. However, the subspecies *Atta sexdens rubropilosa* and *A. sexdens sexdens* were recorded as *A. sexdens*, because *A. s. rubropilosa* is a junior synonym of *A. sexdens* and *A. s. sexdens* is not currently in use (Antwiki, accessed March 15th, 2020: 24, 25). Most of our results are presented as “number of reports” or mentions of the genus or species throughout all papers, instead of the number of papers, because a study can include several species, and they can vary in the origin of the species (e.g., a single study considering species with tropical distribution and with non-tropical distribution).

For species with Neotropical distributions, we tested whether ant subfamilies have been studied proportionally to the diversity of existing recorded species per subfamily. We used the number of species per subfamily reported in Fernández et al. (27). We then performed two Kolmogorov-Smirnov Goodness of Fit tests (28) to compare the number of species per subfamily present in the Neotropics with 1) the observed number of species per subfamily studied in the Neotropics, and 2) the number of species per subfamily studied in the Neotropics regarding behavior. In the papers regarding the behavior of ants with a Neotropical distribution, we recorded whether the behavior was studied 1) in the laboratory, 2) in the field, 3) if it was modeling or simulation, or a combination of those, for each of the species separately. We wrote “NA” in a few cases where we did not have access to the full text. We performed a Fisher’s exact test of independence to check if ants were more often studied in the field than in the laboratory, using the top ten most studied genera of ants in behavioral studies with Neotropical ants. All statistical analyses were performed using R version 4.0.3 (29).

To determine from which countries ants were studied the most in recent scholarly papers, we recorded the country of collection of each species mentioned in each retrieved paper. We used the category ‘lab colony’ when the ants came from laboratory colonies and the authors had not specified the original collecting site in the Methods section. We recorded ‘exotic’ in the country if the ants were collected in a country from which they are non-native (according to https://antmaps.org/ 24, 25), and the report gets assigned as another advancement in the knowledge about the region where the ant is originally from (e.g., a study of *Solenopsis invicta* in Asia was assigned as a Neotropical record, and the country was recorded as “exotic”). Again, results are presented as the number of reports for each country, rather than the number of scientific papers per country, because a single paper can list several countries.

## Results

### General search

Regarding the general search (2015 to 2019), we found a mean of 57388 (range: 370–299000) papers published for the search A (“ant” OR “ants” OR formicidae) across all databases, 53887 (range: 4837–94209) records for the search B (tropic* OR neotropic*) and 10551 (range: 385–35233) for the search C (behav* AND (tropic* OR neotropic*). Search 1 (A&B combined) generated 1833 (range: 54–4200), whereas Search 2 (A&C combined), regarding the behavior of Neotropical ants, generated an average of 2015 records (range: 6–9080). Across all databases, we selectively retrieved 8,966 potentially relevant citations (by combining the results from searches 1 and 2 from all databases except Google Scholar, due to export limits) regarding the behavior of Neotropical ants. After deduplicating, the preliminary count for this search is 5,447 potentially relevant published papers about the behavior of Neotropical ants (**Supplementary Table S1**). This means that approximately 9.5% of the papers published regarding ants, studied at least one aspect of the behavior of Neotropical ants between 2015 and 2019. However, some results might still overlap between searches.

### Targeted search

**Figure 1:**
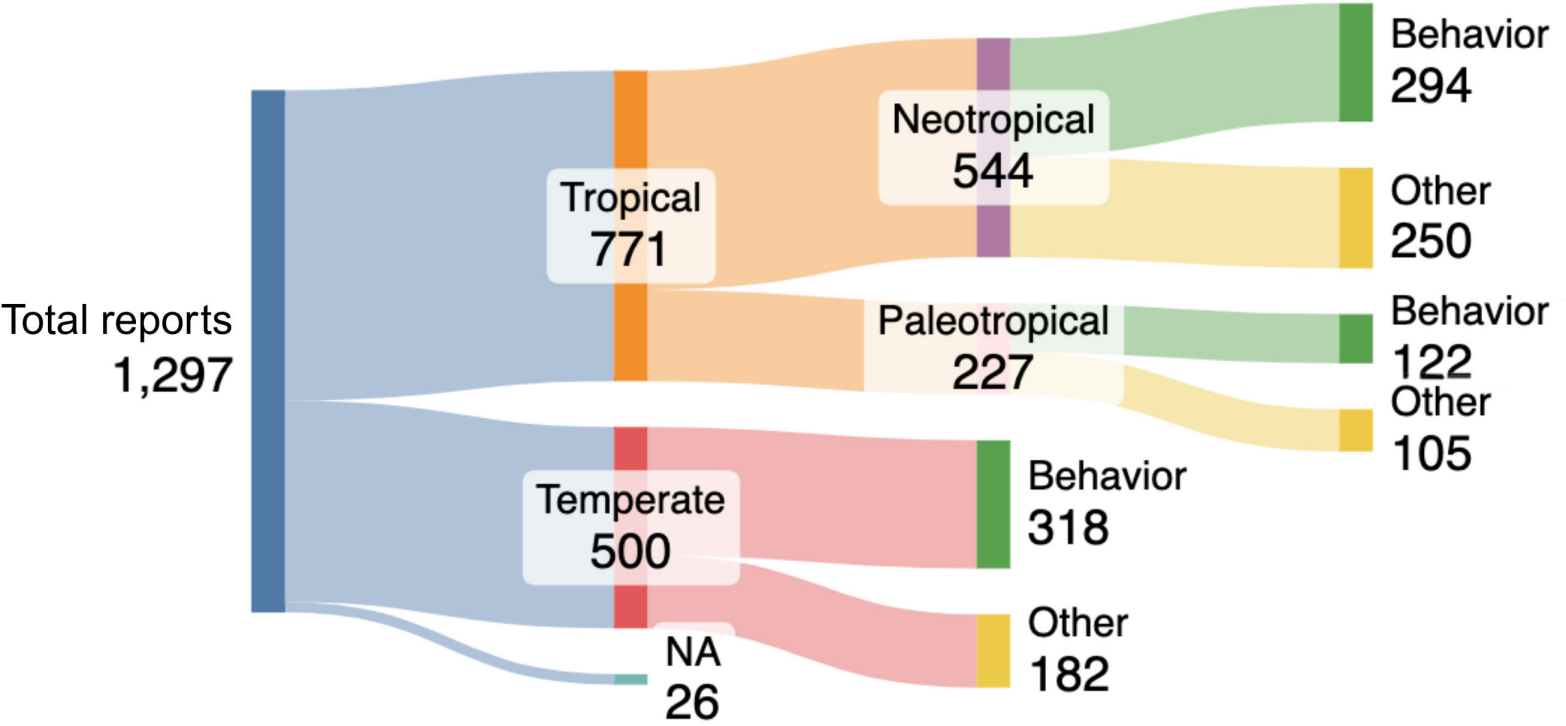
Reports about ants published in 23 scientific journals between 2015 and 2022 were categorized according to the geographical ant distribution, and whether the report included any aspect of ant behavior. NA refers to articles doing theoretical modeling. Reports do not correspond to the number of publications because a single publication can encompass multiple species from different geographical origins.

In the targeted search (2015 to 2022), we found 1297 reports about ants published from our selected journals, of which ~60% were from tropical species, ~38% were from temperate species, and 2% used theoretical models. We found that among all tropical ant reports, 70% pertained to ants from the Neotropics compared to ants from the Paleotropics. In both the Neotropics and the Paleotropics, about half of the reports studied aspects of behavior. In contrast, in temperate regions, reports of ant behavior represent more than 60% of the records. Overall, ~42% reports focused on Neotropical ants, and ~23% included aspects of their behavior (**Figure 1**).

### Most studied taxa (subfamily level)

**Figure 2:**
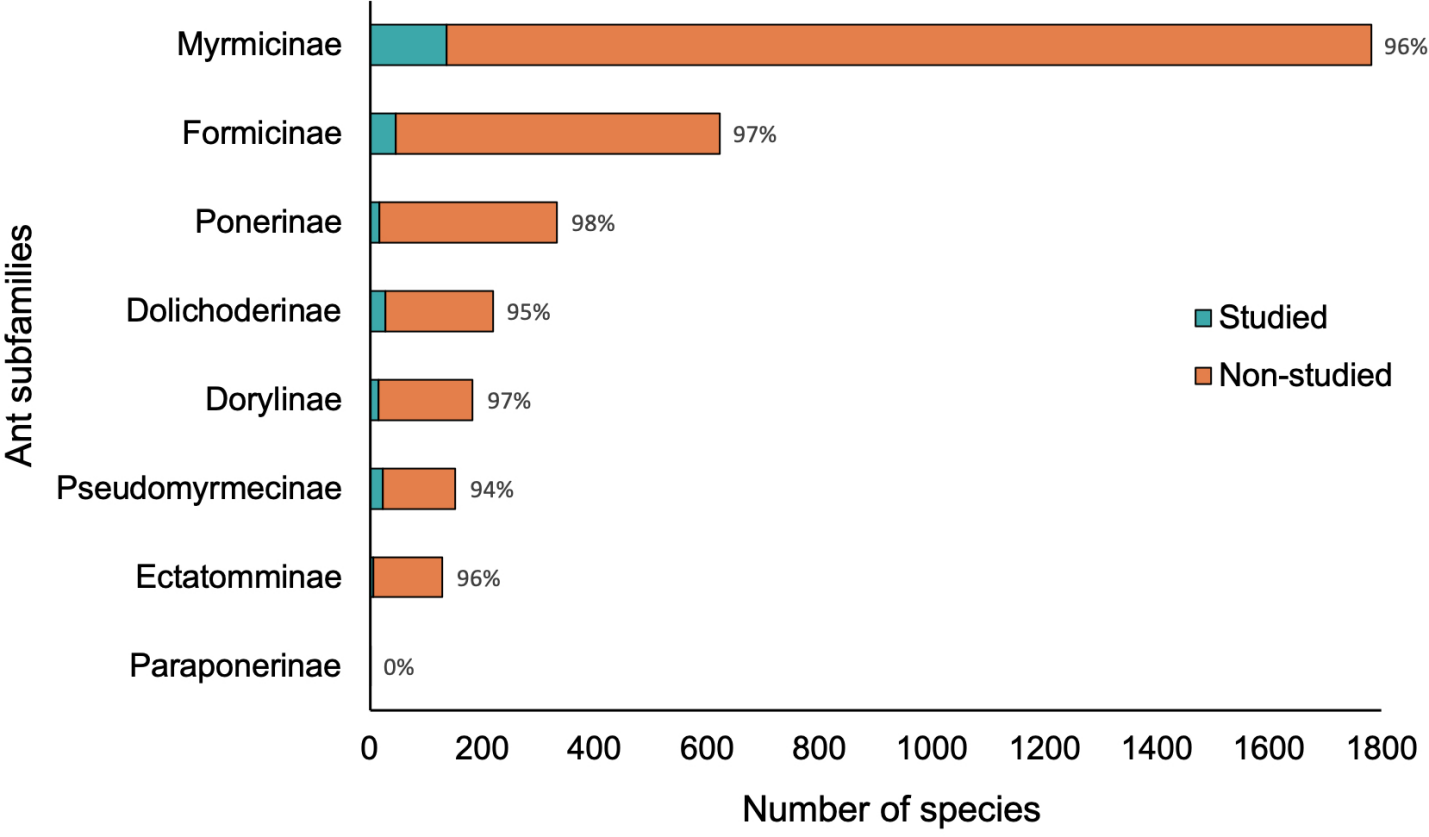
Number of studied species (cyan) compared to the total number of species by subfamily (orange) regarding the behavior of Neotropical ant species (294 reports). The subfamilies Agroecomyrmecinae, Amblyoponinae, Martialinae, and Proceratiinae lack behavioral studies among those dates. Bar labels indicate % of non-studied species.

The existing species diversity per subfamily in the Neotropics was not proportional to the Neotropical species studied (Kolmogorov-Smirnov Goodness of Fit test, D = 0.667, p= 0.037) nor the Neotropical species studied for behavior (Kolmogorov-Smirnov Goodness of Fit, D = 0.75, p= 0.018; **Figure 2**). Scientific publications mention only ~0 to ~18% of the species reported per subfamily for the Neotropics. The only subfamily with full representation was Paraponerinae, which contains only a single species. The species from the Neotropics with behavioral studies cover only ~0 to ~15% of the diversity of each subfamily. Moreover, four subfamilies in the Neotropics lacked studies on the behavior of the ants in the studied period: Agroecomyrmecinae (5 species), Amblyoponinae (68 species), Martialinae (1 species), and Proceratiinae (66 species).

### Most studied taxa (genus level)

**Figure 3:**
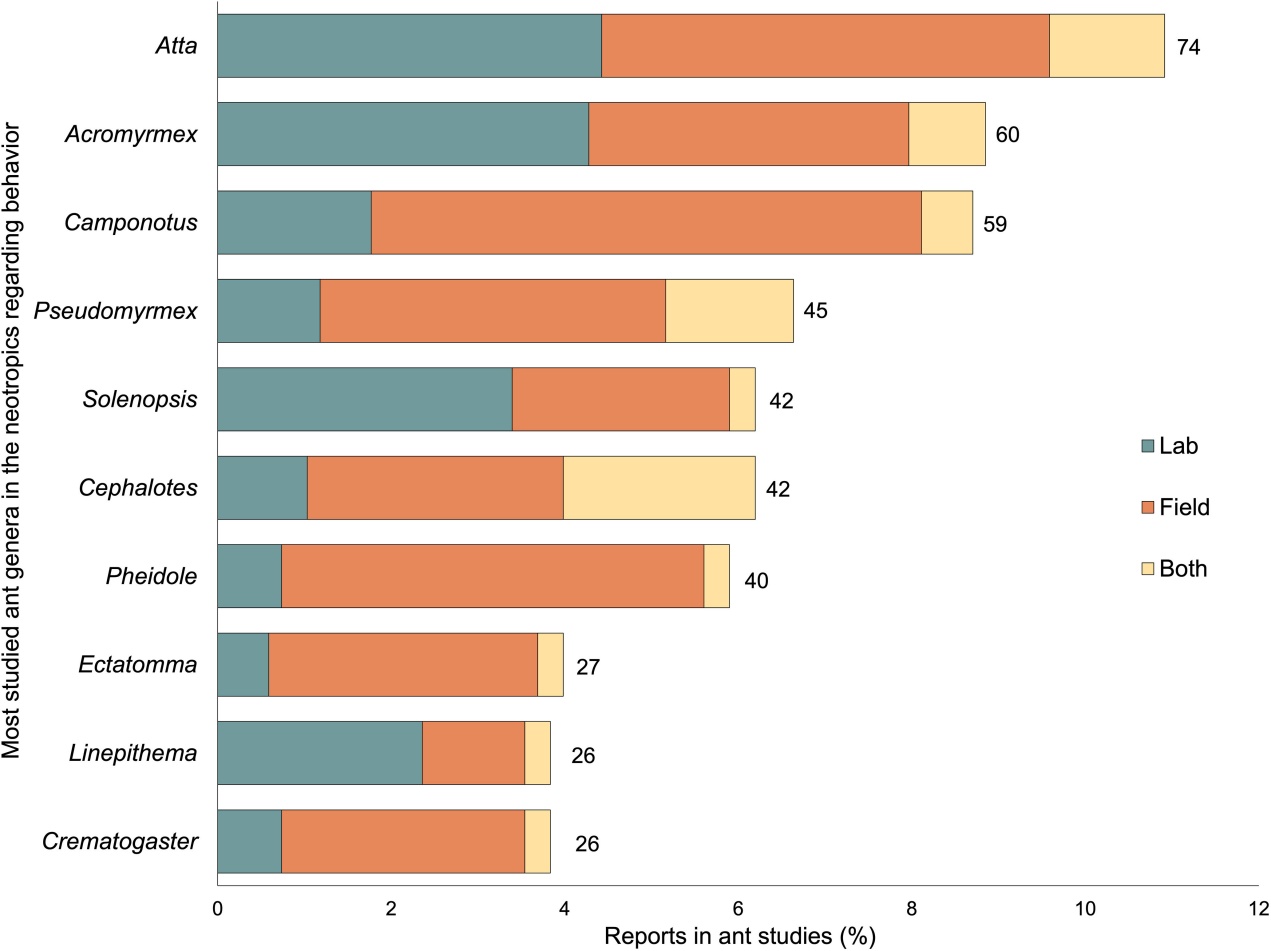
Top 10 most studied ant genera in the Neotropics in papers regarding behavior published between 2015 and 2022 (n = 294 reports), categorized by studies done in the laboratory, in the field, or both. Bar labels indicate the number of genera reports.

The top Neotropical genera in studies of behavior were *Atta*, *Acromyrmex*, and *Camponotus* (**Figure 3**). Leaf-cutter ants (*Atta*, *Acromyrmex*) accounted for about 20% of the reports in studies regarding behavior. Ants were more likely to be studied in the field than in the laboratory (**Figure 3**; Fisher’s exact test; p=0.0005). Within behavioral studies of Neotropical ants, we identified 41 genera that were mentioned less than 10 times (half of them were mentioned 3 times or less) in the retrieved studies. *Atta, Acromyrmex, and Camponotus* remain as the top-studied genera in the tropical and Neotropical region, considering all subjects and not only behavior (**Supplementary Figure S1**). Worldwide, *Camponotus* is the top-studied genus, and *Atta* and *Acromyrmex* remain among the top, along with *Formica* and *Solenopsis*. In temperate regions, *Formica, Lasius*, and *Temnothorax* are among the top genera studied (**Supplementary Figure S1**).

### Most studied taxa (species level)

**Figure 4:**
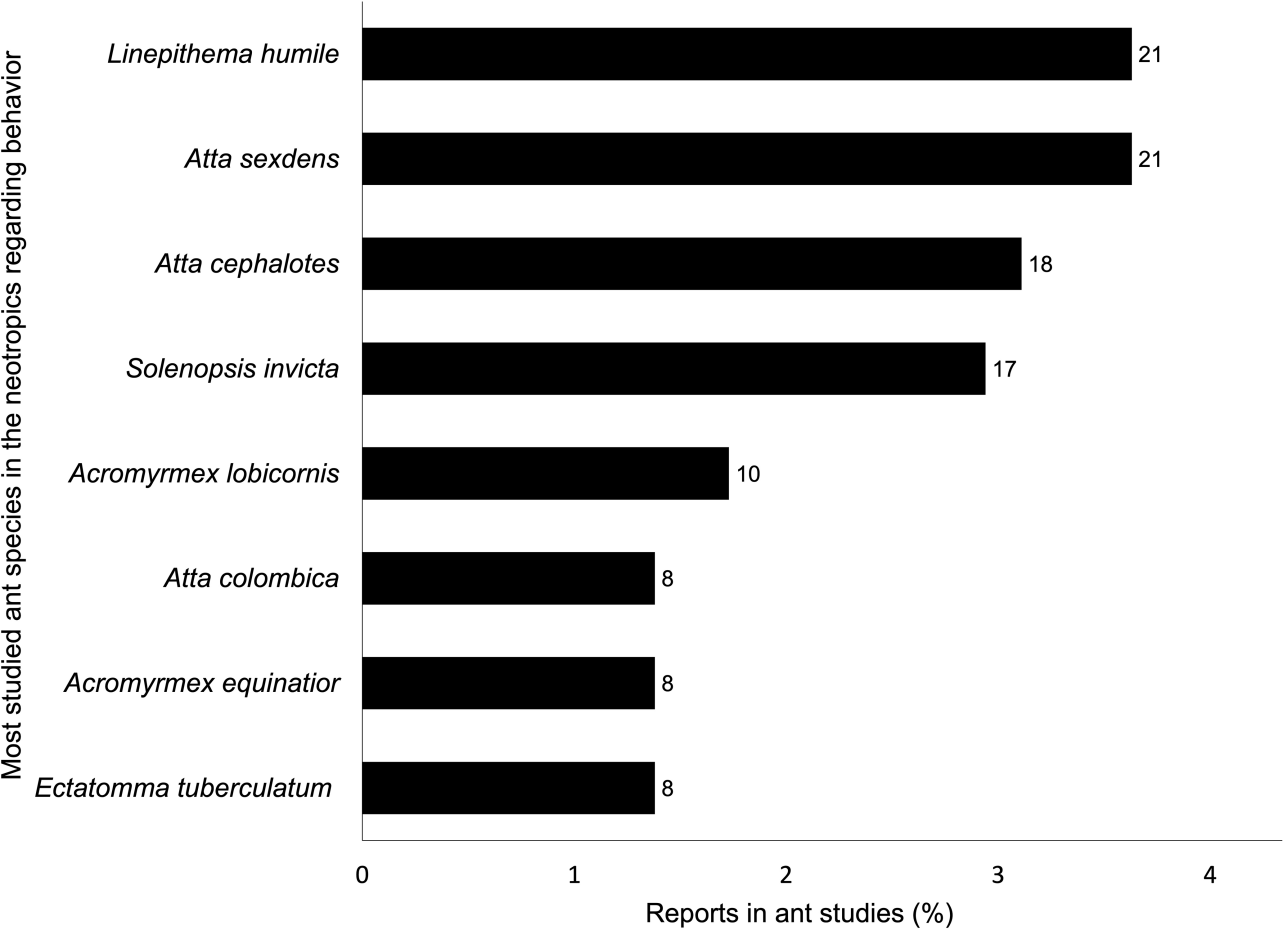
Most studied ant species in the Neotropics in papers regarding behavior (n = 294 reports). Bar labels indicate the number of species reports.

In the Neotropics, 3463 extant ant species have been reported (27), but only ~10% of those species (358 species) have been studied in recent years. Additionally, the behavior of less than 8% of the extant species (273 species) has been studied recently. Regarding the behavior of Neotropical ant species, *Linepithema humile*, *Atta sexdens, Atta cephalotes*, and *Solenopsis invicta* are among the most studied species (**Figure 4**). Interestingly, more than half of the studied species were recorded a single time across all examined studies. Considering all subjects, the same species (*S. invicta*, *L. humile*, *A. sexdens*, *A. cephalotes, and A. lobicornis*) remain among the most common in the tropics and Neotropics, but worldwide, the most studied *Acromyrmex* is *Acromyrmex echinatior* (**Supplementary Figure S2**). *Lasius niger* and *S. invicta* are the species with the most recent reports in the world. In the temperate zones, the most studied species is *L. niger* followed by *Myrmica rubra and Temnothorax rugatulus* (**Supplementary Figure S2**).

### Laboratory vs. field

**Figure 5:**
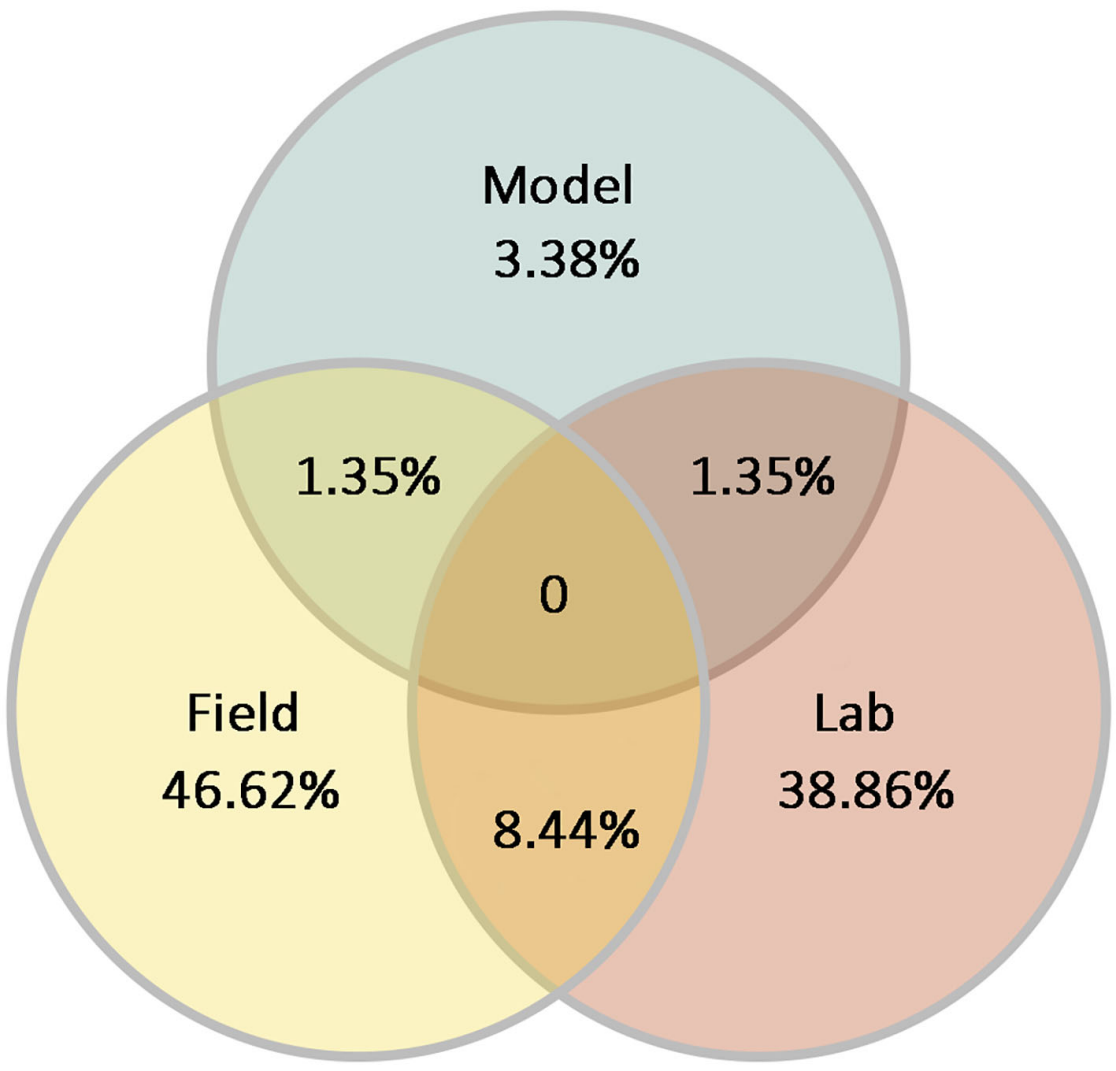
Percentage of reports about the behavior of Neotropical ants detailing whether they were performed in the laboratory, in the field, using simulations or modeling, or any combination of them (n = 294 reports).

Considering all reports on the behavior of Neotropical ant species, most studies were conducted either in the lab or in the field, and fewer studies combine both approaches. Also, a small percentage are based solely on modeling, and even fewer studies integrated lab or field approaches with modeling (**Figure 5**).

### Country of collection

**Figure 6:**
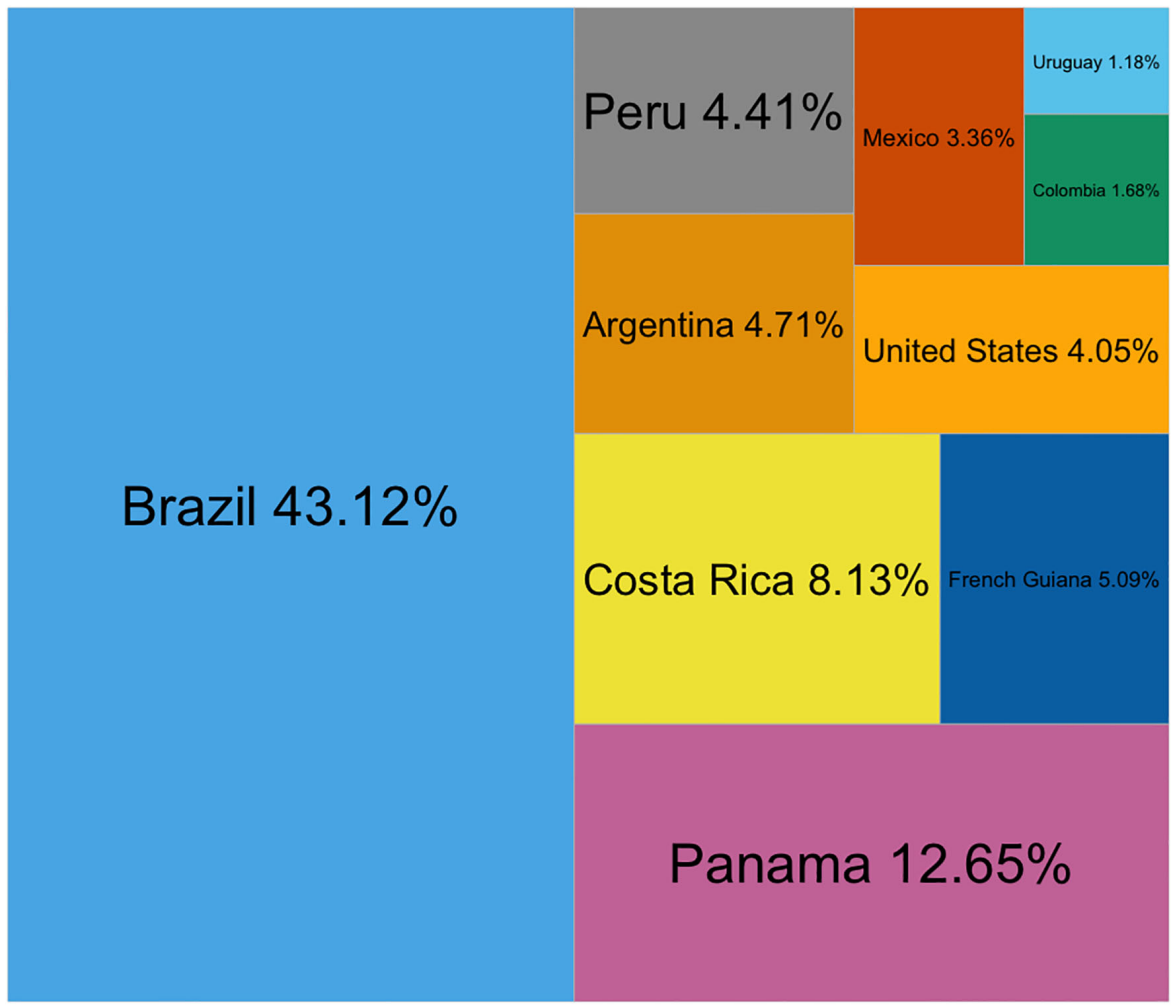
Countries where ants with a Neotropical distribution have been collected to study their behavior between 2015 and 2022 (n = 294). The category ‘exotic’ was 7.4%, while ‘lab colony’ and ‘global’ comprised 1.35% and 0.67% of the mentions, respectively.

In recent years, Brazil is the worldwide leader regarding the number of ant species collected for studies, followed by the United States and Panama (**Supplementary Figure S3A**). In the tropics, the four countries with the most reports were Brazil, Panama, Mexico, and Costa Rica (**Supplementary Figure S3B**). In temperate zones, most ants were from the United States, Spain, and Germany (**Supplementary Figure S3C**). In the Neotropics, Brazil accounts for the majority of the reports, followed by Panama. The third place was occupied by Costa Rica and Mexico (**Supplementary Figure S3D**). Brazil, Panama, and Costa Rica were the three countries where the most ants have been collected for behavioral studies (**Figure 6**). Nicaragua, Honduras, Guatemala, Belize, and El Salvador did not have reports for those years.

## Discussion

In this study, we tested whether scientific publications examining Neotropical ant behavior reflected the diversity of Neotropical ants. Our results indicate that recent studies on ant behavior in the Neotropics focus predominantly on four species, which represent less than 0.15% of the total Neotropical ant diversity. Moreover, we found that recent ant behavior studies encompass less than 8% of the Neotropical ant biodiversity. Our results also revealed a geographical bias, whereby Central American countries such as Nicaragua, Honduras, Guatemala, Belize, and El Salvador had not recently produced any ant studies in the scrutinized journals, despite their high biodiversity.

To date, ~26% (3463 species) of the extant ant species worldwide are considered Neotropical (27), and this number is continuously increasing. In the targeted search, we found that ~42% of the reviewed papers on ants worldwide examined Neotropical ants, with half of them (~23%) focusing on their behavior, which is an overrepresentation compared to studies on Paleotropical ants. However, this apparently large percentage of reports included only ~10% (358 species) of the Neotropical ant species, and recent behavioral information exists for fewer than 8% of the species (273 species). Considering all scientific publications, the percentage of papers studying Neotropical ant behavior might be even lower than our numbers indicate, as shown by our results in the general search compared to the targeted search: the percentage of papers studying at least one aspect of Neotropical ant behavior between 2015 and 2019 decreased from ~23% using the general research to ~9% when using the targeted research. In sum, we have studied the behavior of only a small fraction of the Neotropical ant species in recent years, regardless of the estimation method. Moreover, we found that the examined scientific publications studying Neotropical ants or the behavior of Neotropical ants include only ~15% of the number of Neotropical species per subfamily (ranging from ~0 to ~15% of the species reported per subfamily for the Neotropics).

We documented that four species encompass the bulk of research about Neotropical ant behavior: the leaf-cutter ants *A. sexdens* and *A. cephalotes* (Myrmicinae), the Argentine ant *L. humile* (Dolichoderinae), and the fire ant *S. invicta* (Myrmicinae). Leaf-cutter ants have a widely recognized role in Neotropical ecosystems as soil modifiers, fertilizers and seed dispersers (see review in 30). However, they are also among the most economically damaging Neotropical ant species, causing substantial losses in several plantations and gardens (22). They affect a variety of crops, including coffee (*Coffea arabica* L.), cocoa (*Theobroma cacao* L.), citrus (*Citrus* spp.), cassava (*Manihot esculenta* Crantz), maize (*Zea mays* L.), cotton (*Gossypium hirsutum* L.), and plantations of conifers and *Eucalyptus* (see 30). Therefore, it is not surprising that they are among the most studied ants in the Neotropics. The other top two studied species are invasive in several countries. *L. humile* is a South American ant that became an invasive species in the United States, the Mediterranean, Japan, and New Zealand due to human introduction (Antwiki, accessed 5 May 2023: 24, 25). Finally, *S. invicta*, the red imported fire ant, is also one of the most damaging invasive species, causing an economic impact in the U.S. estimated at more than US $6,3 billion per year (31). An alarming consequence of concentrating most research on a few species within the Neotropics is that around 85% of the Neotropical ant species have not been studied recently, which may contribute to reduced ability to prevent or control new possible invasions, difficulties to reveal evolutionary patterns, understudied roles in Neotropical ecosystems and interactions with other species that can be beneficial for humans, and increased accuracy of phylogenetic reconstructions (32, 33).

Moreover, damage from ant invasions incurred costs of at least US$10.95 billion between 1930 and 2020 worldwide, in addition to approximately US$40 billion in potential costs. This number could be an underestimation because 75% of the invaded countries lack cost reports (21). If we studied more ant species, there would be a higher probability of avoiding these costs, as most of these expenses were related to post-invasion management rather than prevention (21). Furthermore, studying behaviors such as habitat selection, interactions with other species, and the dispersal of insect species (34) is key to developing management solutions. For instance, the South American Tawny crazy ant *Nylanderia fulva* has displaced native species in the United States and caused agricultural and biological damage in Colombia (Antwiki, accessed 5 May 2023; 24, 25), Costa Rica, and Panama (35). To manage this invasive species, the study of animal behavior has become crucial. Ultimately, behavioral traits are primarily responsible for the successful invasion of a new habitat (36). Therefore, efforts to broaden the number of ant species represented in behavioral studies are essential for achieving a more general understanding of ant ecology and behavior, and are key to finding solutions to invasions as well as successful conservation measures.

We identified a gap in publications studying the subfamilies Agroecomyrmecinae, Amblyoponinae, Martialinae, and Proceratiinae, which is not surprising given their rarity or limited species diversity. However, these ant subfamilies hold key insights into their evolutionary, ecological, and morphological aspects that merit closer scientific attention. Agroecomyrmecinae comprises only two extant species in the Neotropics. One of them, known as “Armadillo Ants,” has a shield-like head (37, 38). Despite the broad geographical distribution of Armadillo Ants (from México to Peru, Brazil, and Surinam), they are cryptic and uncommon (39). Similarly, the subfamily Martialinae contains only one species, a cryptic, eyeless, subterranean predator that has been collected only in the Amazon rainforest of Brazil (40). The subfamilies Amblyoponinae and Proceratiinae are more numerous in species and are distributed throughout the Neotropical region, but they are also considered rare compared to other ant subfamilies. Amblyoponinae comprises 16 reported species for the Neotropics, and consists also of cryptic predatory ants with reduced or absent eyes (27). They specialize in centipedes living on the forest floor, and adults can feed on the larval hemolymph, probably when prey availability is limited (41), giving them the name of “Dracula Ants” (42). Finally, Proceratiinae contains 25 species in the Neotropics (27). All are small and cryptic species also with reduced or absent eyes and specialized in arthropod eggs (see 43). In general, these four subfamilies are predatory, cryptic ants with relatively few species and few representatives in collections, and have been proposed as basal when compared to related clades (37, 40, 42, 44, 45). Examining relict subfamilies, such as Martialinae, is crucial for unraveling taxonomic relationships and the evolution of morphological and behavioral characteristics present in more derived taxa (46). At the genus level, some genera with few records are poorly known. These genera may be geographically restricted or less frequently encountered in the field, which could further contribute to their underrepresentation in behavioral research.

The most species-rich ant subfamilies also have a small fraction of their species examined in recent studies in the Neotropics. For instance, the subfamily Myrmicinae (1782 species) comprises about 51% of all ant species reported for the Neotropics (27); yet only around 10% of them appeared in our search as the main study organism, and even fewer appeared in studies about behavior (8%). Therefore, our findings show that the scholarly published records sampled in this study do not fairly represent the vast Neotropical ant diversity. This finding is concerning because studying an extensive array of the existing species within a taxon significantly facilitates achieving a more complete understanding of its phylogenetic relationships (32, 33), and therefore the evolution of morphological, physiological, and behavioral traits (e.g., 47–49).

Regarding the country of collection, it is not surprising that Brazil holds the top position globally and within the tropical and Neotropical region, as it is the fifth largest country in the world (https://www.un.org/esa/earthsummit/brzil-cp.htm and https://data.un.org/) and the most biodiverse country in the Americas (50). Its geographical location, large size, and diversity of ecosystems likely contribute to its leading role. In recent years, Brazil has also been at the forefront of Neotropical ant physiology studies (51). There is also a long tradition of studying myrmecology in Brazil (52), which hosts one of the world’s most important myrmecology conferences and maintains a core faculty of myrmecologists who train young scientists (53, 54). However, given Brazil’s vast territory, it is likely that many regions and ant species remain understudied, particularly in terms of behavior. Moreover, we found that smaller countries such as Panama and Costa Rica have proportionally more studies per unit area compared to bigger countries such as Brazil. Panama and Costa Rica are also highly biodiverse, but have vastly reduced geographic extent and research funding compared to Brazil. However, the two countries share a long history of international institutions and the presence of political stability that may have facilitated research projects by local or foreign scientists (4, 55, 56). Accordingly, Brazil, Panama and Costa Rica along with Mexico have led scientific productivity in ecological research in the tropics, in which researchers from Brazilian institutions author most studies produced in Brazil. In contrast, the studies about ants from Costa Rica and Panama are more likely led by foreign researchers, which might be explained by the presence of the Organization for Tropical Studies in Costa Rica, and the Smithsonian Tropical Research Institute in Panama (4). Similarly, in the field of ant physiology, Brazil, Panama, and Costa Rica (tied with French Guiana) are leaders as countries of collection (51). Unfortunately, other megadiverse Neotropical countries, likely facing limited research funding, showed no records of ants collected for behavioral studies. There is widespread concern that countries with high biodiversity but low income lack the necessary resources to study and monitor their wildlife (57). As our quantification is related to the country of origin of the species, rather than the authors’ country of affiliation, the lack of funding for research may not be a sufficient explanation for their absence in our records. It is possible that other socioeconomic factors, political instability, internal conflicts, or processes related to research or export permits also contribute to the relatively few studies in those countries. In some cases, the presence of a principal investigator with expertise in a particular genus or group can shape the trajectory of student projects, founding long-standing research lines in specific countries (58).

## Conclusions

Among the reasons why ant diversity is unevenly studied, we identified that species of economic importance, either in agriculture or as invasive species, are more likely to be the focus of behavioral studies. Additionally, cryptic habits and low abundance can limit opportunities for their observation, leading to fewer behavioral studies than expected. On the other hand, long-standing research traditions on species that are more abundant, easier to find, or better known taxonomically, may also reinforce taxonomic biases. The presence of well-established researchers with expertise in a particular genus or group can shape the trajectory of young researchers (58) and further concentrate research efforts on particular species and countries. Moreover, we observed that countries with prominent research institutions in the Neotropics are more likely to produce scientific studies on the behavior of Neotropical ants.

Several ant species are ubiquitous, abundant, and relatively easy to find (59). Ants are diverse not only in the number of species but also in life history, ecology, and behavior. For several genera, taxonomic keys are available, and identification using morphological characters is reliable. Some species can be kept in captivity, and others can be studied in the field in abundant numbers. Yet, our study reveals that four species dominate recent studies about ant behavior in the Neotropics, which constitute less than 0.15% of the ant species in the Neotropics. We also found that 50% of the studied species were mentioned once or twice in studies regarding behavior. Moreover, recent studies on ant behavior cover less than 8% of the Neotropical ant biodiversity. Assessing the behavior of a diverse group of ants allows us to understand evolutionary trends, novelties and adaptations, and to shed light on the evolution of sociality. Our results revealed a geographical inequity in the study of ants within the Neotropical region, given that highly biodiverse countries such as Nicaragua, Honduras, Guatemala, Belize, and El Salvador lack recent studies focused on ant behavior. Further studies should address whether the Paleotropics exhibit a similar bias.

Other groups of arthropods are further behind, due to difficulties in taxonomic identification and sampling. Further research could investigate whether these patterns are the result of historical trends in ant research. Our study highlights the crucial role of well-established research centers in advancing scientific knowledge, calls for more equal research and funding opportunities in poorly studied countries, highlights the need for a more diverse approach to the study of ant behavior and encourages researchers to study relatively unknown species of ants to fill the gap in the current knowledge about ant biology.
