# Supplementary material for: State of the ant: how broad is our recent knowledge of Neotropical ant behavior?
Source: Front Insect Sci. 2025 Sep 10;5:1613264. doi: 10.3389/finsc.2025.1613264 (PMC12459118; doi:10.3389/finsc.2025.1613264)
Supplement: Supplementary file 1 [file Supplementaryfile1.docx]

**Supplementary material**

**
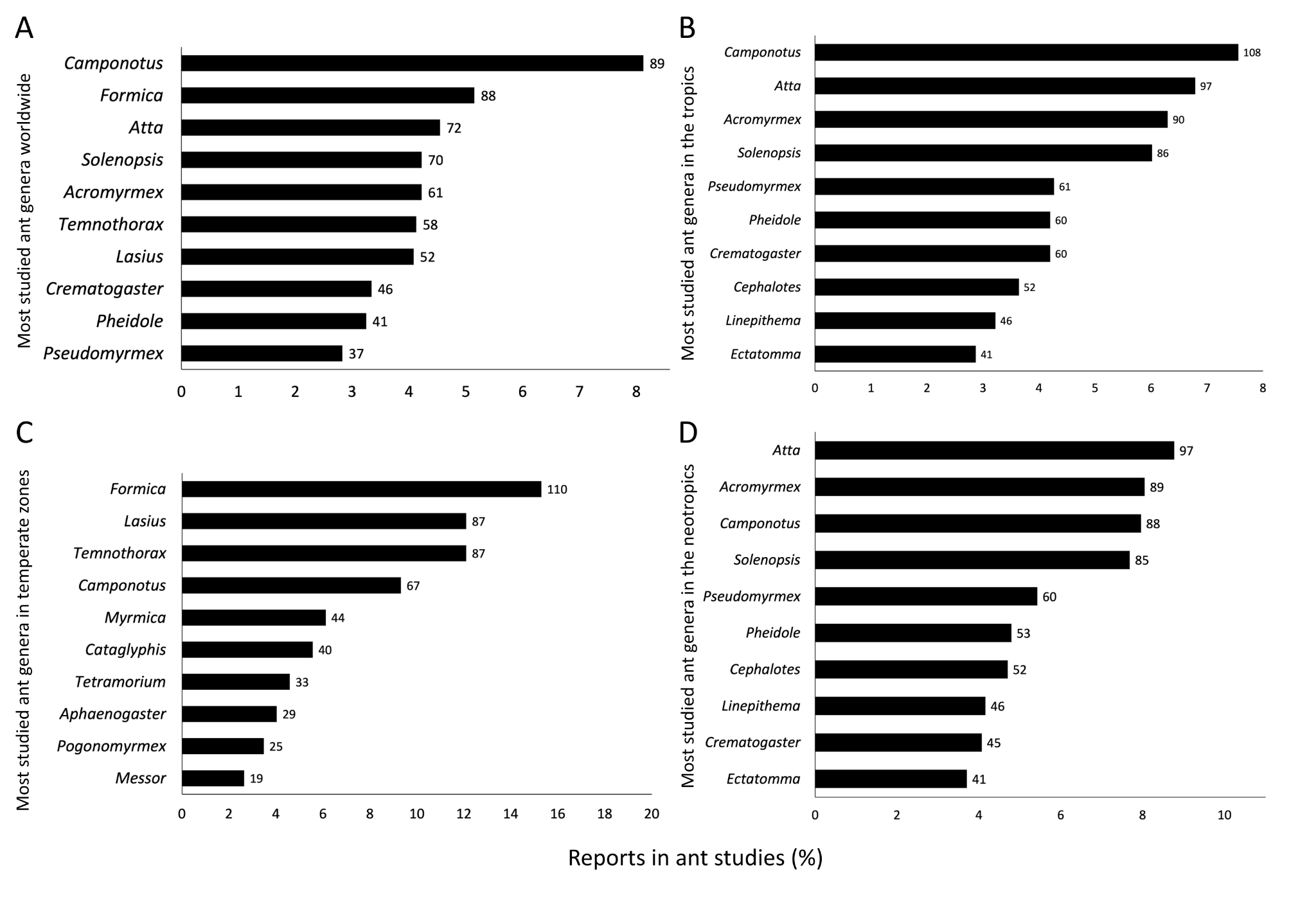
**

**Figure S1**. Most studied ant genera in articles published between 2015 and 2022: A) worldwide; B) in the tropics; C) in temperate zones; D) in the Neotropics. Number of reports for each region are available in Fig. 1.

**
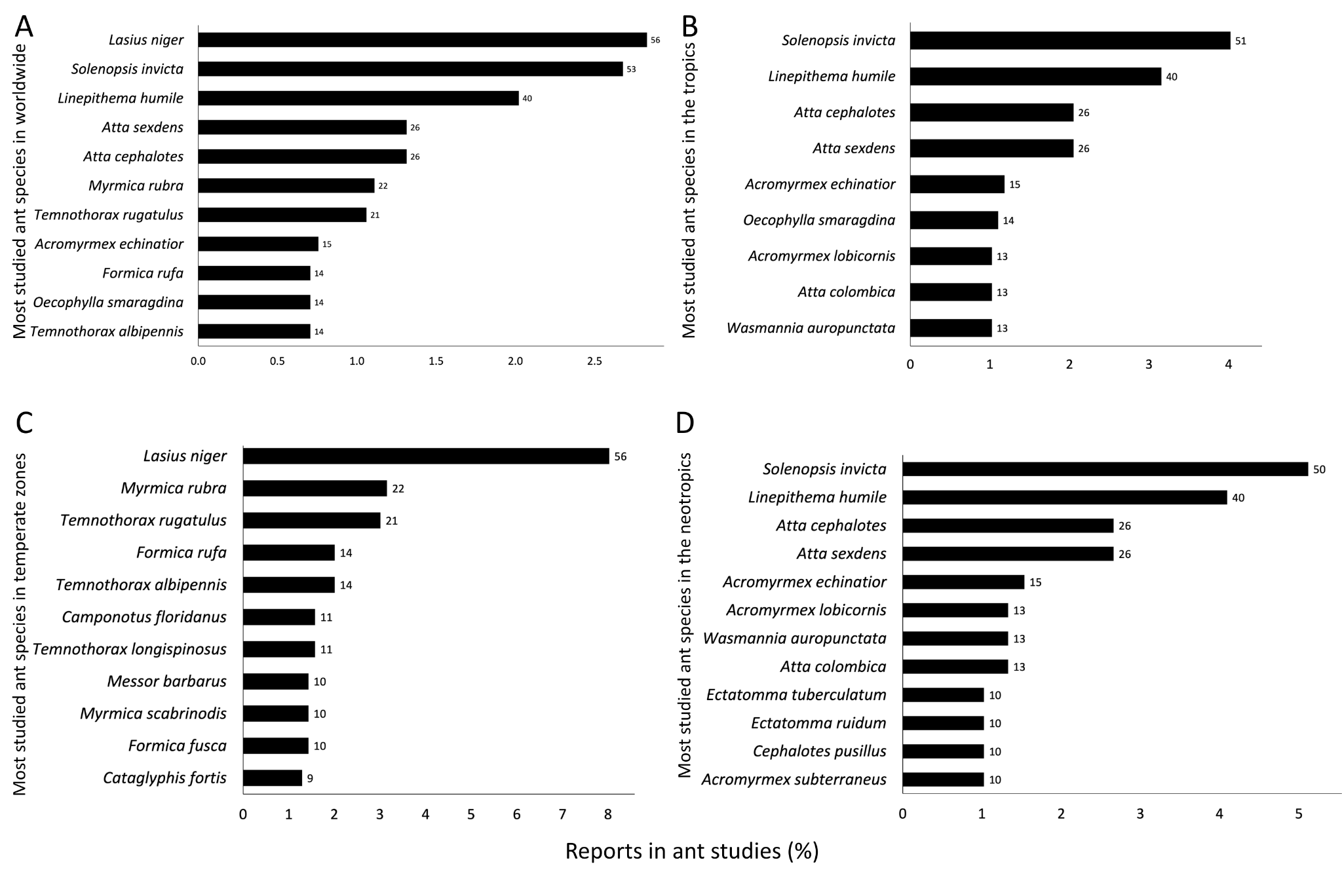
**

**Figure S2**. Most studied ant species in articles published between 2015 and 2022: A) worldwide; B) in the tropics; C) in temperate zones; D) in the Neotropics. Number of reports for each region are available in Fig. 1.


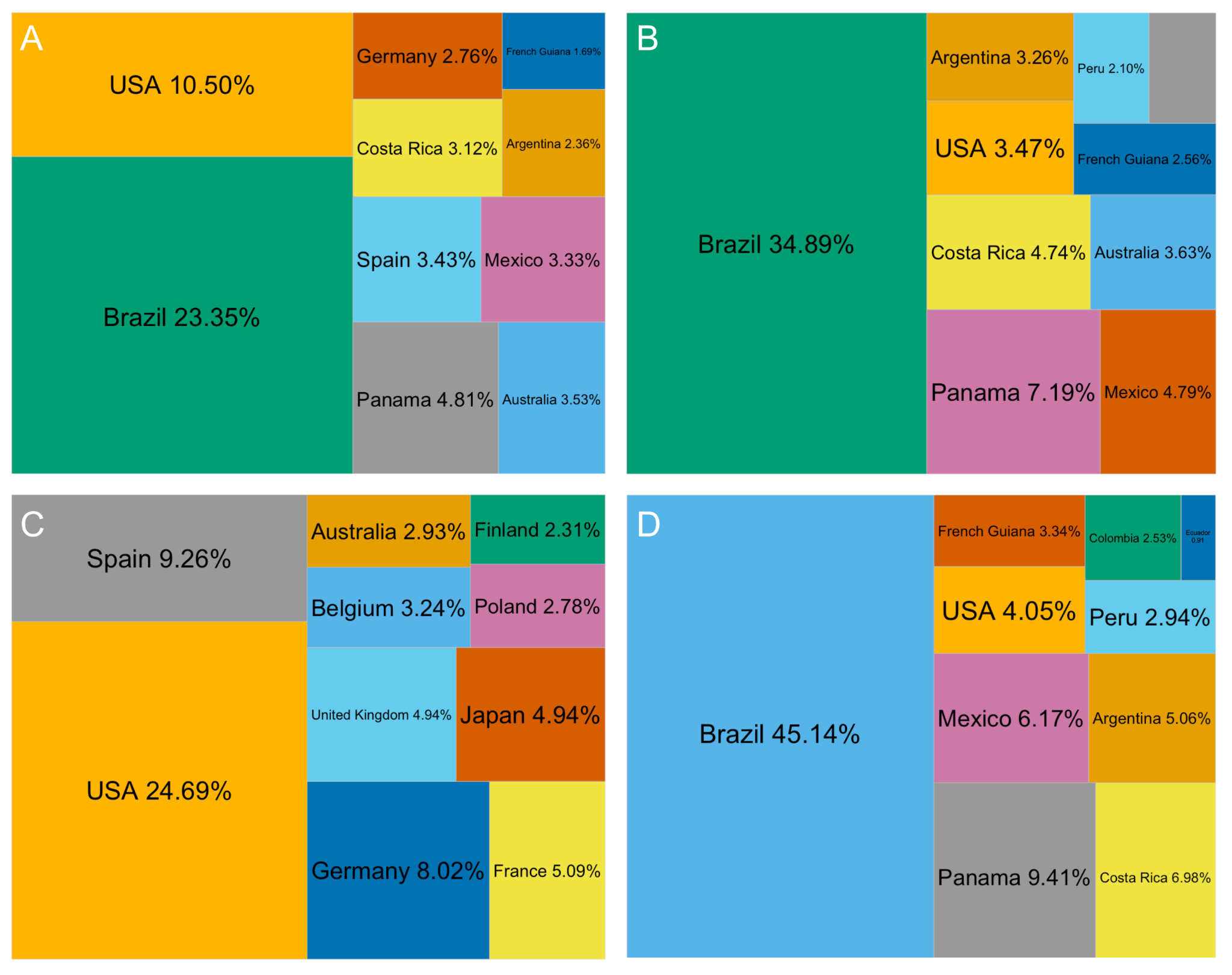


**Figure S3**. Top 10 countries where ants have been collected the most in studies published between 2015-2022: A) worldwide; B) in the tropics; C) in temperate zones; D) in the Neotropics.

**Table S1.** Results for Searches 1 (ant” OR “ants” OR formicidae) AND (tropic* OR neotropic*) and 2 (“ant” OR “ants” OR formicidae) AND behav* AND (tropic* OR neotropic*) across 6 databases. Curated Total denotes numbers after culling and deduplicating/merging results between searches 1 and 2. Some results might still overlap both searches.

|  | *Web of Science* | *Zoological Record* | *SciELO* | *PubMed* | *ProQuest PACD* | *Google Scholar* | **RAW TOTAL** | **CURATED TOTAL** |
| --- | --- | --- | --- | --- | --- | --- | --- | --- |
| Search 1: (“ant” OR “ants” OR formicidae) AND (tropic* OR neotropic*) | 975 | 1,480 | 54 | 417 | 3,871 | *4,200 | 10,997 | 4,679 |
| Search 2: (“ant” OR “ants” OR formicidae) AND behav* AND (tropic* OR neotropic*) | 189 | 615 | 6 | 137 | 2,062 | *9,080 | 12,089 | 2,676 |
| **DATABASE TOTAL** | 1,164 | 2,095 | 60 | 554 | 5,933 | *13,280 | **23,086** | **5,447** |

*Not retrieved due to database limits.

For the general search we searched in 6 databases (Web of Science, Zoological Record, SciELO, PubMed, ProQuest Databases and Google Scholar) and performed 3 baseline searches on each database: A) (“ant” OR “ants” OR formicidae); B) (tropic* OR neotropic*) and; C) (behav* AND (tropic* OR neotropic*) (Table S1). We then performed 2 complex search strings: 1) searches A&B combined and 2) searches A&C combined.

**Table S2.** Number of papers about ants, about the behavior of Neotropical ants, and the total number of published papers in 23 scientific journals from 2015 and 2022. Journal’s impact factors (from 2019) were recorded from each journal's homepage, except those indicated with an asterisk.

| Journal | Journal’s  impact factor |  | Number of papers about ants | Number of papers about the behavior of Neotropical ants | Total published papers |
| --- | --- | --- | --- | --- | --- |
| Plos One | 2.740 |  | 141 | 29 | 109067 |
| Insectes Sociaux | 1.418 |  | 140 | 44 | 358 |
| Scientific Reports | 3.998 |  | 95 | 12 | 96179 |
| Myrmecological News | 2.558 |  | 69 | 8 | 96 |
| Sociobiology | 0.690 |  | 66 | 35 | 395 |
| Journal of Experimental Biology | 3.014 |  | 61 | 11 | 3244 |
| Proceedings of the Royal Society B | 4.637 |  | 56 | 13 | 2988 |
| Behavioral Ecology and Sociobiology | 2.277 |  | 46 | 9 | 1047 |
| Biotropica | 2.090 |  | 39 | 15 | 497 |
| Animal Behaviour | 2.689 |  | 34 | 12 | 1513 |
| Naturwissenschaften | 2.090 |  | 28 | 4 | 473 |
| Journal of Insect Behavior | 0.991 |  | 27 | 10 | 262 |
| PNAS | 9.412 |  | 25 | 5 | 18685 |
| Behavioral Ecology | 2.761 |  | 24 | 8 | 1066 |
| Biology Letters | 2.869 |  | 19 | 1 | 1137 |
| Entomologia Experimentalis et Applicata | 1.696 |  | 14 | 3 | 612 |
| Revista de Biologia Tropical | 0.703 |  | 14 | 4 | 853 |
| Ethology | 1.467 |  | 9 | 1 | 566 |
| American Naturalist | 3.744 |  | 7 | 4 | 820 |
| Current Zoology | 2.351 |  | 6 | 0 | 417 |
| Science | 41.845* |  | 6 | 0 | 7485 |
| Journal of Ethology | 1.400 |  | 1 | 0 | 238 |
| Nature | 42.779 |  | 1 | 0 | 8272 |
| Adaptive Behavior | 0.929 |  | 0 | 0 | 157 |
| Advances in the Study of Behavior | 4.410* |  | 0 | 0 | 35 |
| Behaviour | 1.055* |  | 0 | 0 | 356 |
| Behavioural processes | 1.846 |  | 0 | 0 | 1039 |

*Impact factors retrieved from: https://www.google.com/search?q=science+impact+factor&oq=Science+impact+factor&aqs=chrome.0.69i59j35i39j0i131i433j69i61l3j69i65l2.1988j0j9&sourceid=chrome&ie=UTF-8 (Science), https://www.resurchify.com/all_ranking_details_2.php?id=2268 (Advances in the Study of Behavior) and https://www.scijournal.org/impact-factor-of-behaviour.shtml (Behaviour).
